# Supplementary material for: Comparative Analysis of Maternal Colostrum and Colostrum Replacer Effects on Immunity, Growth, and Health of Japanese Black Calves
Source: Animals (Basel). 2024 Jan 22;14(2):346. doi: 10.3390/ani14020346 (PMC10812718; doi:10.3390/ani14020346)
Supplement: Supplementary file 1 [file animals-14-00346-s001.zip › Figure S1.pdf]

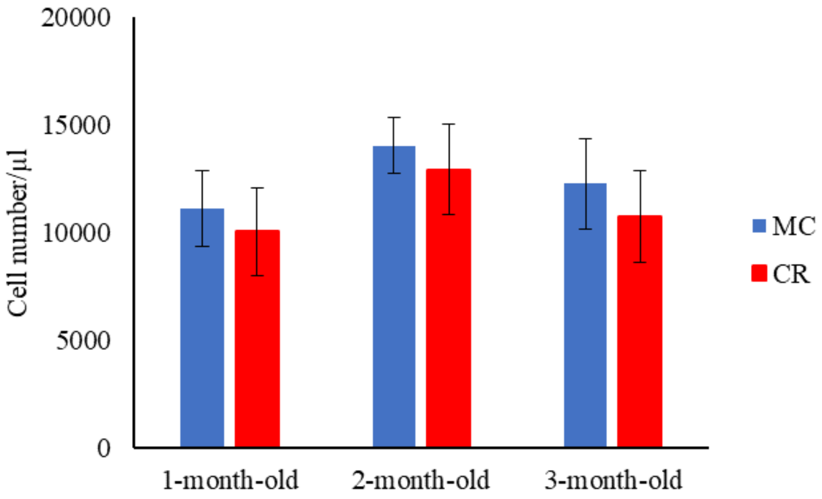

Figure S1: White blood cells (WBC) populations during one, two, and three-month-old in MC and CR group. No significant differences were observed between MC and CR group for WBC population during the period of study. Data are presented as the mean ± SD
