# Supplementary material for: Comparative Analysis of Maternal Colostrum and Colostrum Replacer Effects on Immunity, Growth, and Health of Japanese Black Calves
Source: Animals (Basel). 2024 Jan 22;14(2):346. doi: 10.3390/ani14020346 (PMC10812718; doi:10.3390/ani14020346)
Supplement: Supplementary file 1 [file animals-14-00346-s001.zip › Table S1&S2.pdf]

Table S1: Daily feeding during pregnancy and after pregnancy for mother cow

Period	Hay (kg)	Concentrated food (kg)	TDN (%)	CP (%)
maintenance	6	1	113	96
end of pregnancy	6	2.5	112	94
postpartum	6	1	141	133

Table S2: Monoclonal Antibodies information for flowcytometry and phagocytosis

Monoclonal Antibody	Specificity Marker	Clone	Isotype	Fluorochrome	Dilution
Anti CD4	CD4	ILA11A	IgG2a	FITC	200
Anti CD8	CD8	CC63	IgG2a	HiLyte™ Fluor 555	200
Anti γδ	γδ	GB21A	IgG2b	HiLyte™ Fluor 647	200
Anti IgM	IgM	BIG73A	IgG1	HiLyte™ Fluor 555	100
Anti MHC II	MHC class II	TH14B	IgG2a	HiLyte™ Fluor 647	200
Granulocyte	IgM	CH138A	IgM	HiLyte™ Fluor 555	100
